# Nivolumab plus ipilimumab versus the EXTREME regimen in recurrent/metastatic squamous cell carcinoma of the head and neck: a cost-effectiveness analysis

**DOI:** 10.1038/s41598-024-57277-7

**Published:** 2024-03-21

**Authors:** Dongmei Ye, Xueyan Liang, Xiaoyu Chen, Yan Li

**Affiliations:** 1 Department of Pharmacy, Guangxi Academy of Medical Sciences and the People’s Hospital of Guangxi Zhuang Autonomous Region, Nanning, Guangxi People’s Republic of China; 2 Phase 1 Clinical Trial Laboratory, Guangxi Academy of Medical Sciences and the People’s Hospital of Guangxi Zhuang Autonomous Region, Nanning, Guangxi People’s Republic of China

**Keywords:** Nivolumab, Ipilimumab, Head and neck squamous cell carcinoma, Cetuximab, EXTREME, Cancer immunotherapy, Head and neck cancer

## Abstract

In the CheckMate 651 study, nivolumab plus ipilimumab versus EXTREME (cisplatin/carboplatin + cetuximab + fluorouracil) regimen was compared for effectiveness. It is not known whether these immunotherapy agents are cost-effective for recurrent or metastatic squamous cell carcinomas of the head and neck (R/M SCCHN). The purpose of this study was to compare the cost-effectiveness of nivolumab plus ipilimumab with EXTREME in the first-line setting from the standpoint of third-party payers in the United States. The projecting of costs and outcomes over 15 years was done using a three-state partitioned survival model discounted by 3% per year. Long-term extrapolation of CheckMate 651 was used to model progression-free survival and overall survival (OS). The incremental net health benefit (INHB), incremental net monetary benefit (INMB), quality-adjusted life years (QALYs), and incremental cost-effectiveness ratio (ICER) were calculated. The uncertainty and stability of the model were accounted for via one-way and probabilistic sensitivity analyses. As compared with nivolumab plus ipilimumab, EXTREME was associated with an increase of 0.154 life-years and 0.076 QALYs, as well as a cost increase of $572 per patient. The corresponding ICERs were $7545/QALY along with the values of INMB and INHB were $113,267 and 0.076 QALYs, respectively, at a willingness to pay (WTP) threshold of $150,000/QALY. The probability of nivolumab plus ipilimumab being cost-effective was > 99% in patients with combined positive score (CPS) ≥ 1, CPS 1–19, or CPS ≥ 20. Moreover, hazard ratio for OS and body weight were the most sensitive parameters for the model. According to sensitivity analyses, these results were generally robust. In overall populations with R/M SCCHN, the EXTREME regimen is cost-effective compared with nivolumab plus ipilimumab. Given a WTP threshold of $150,000 per QALY, the probability of the EXTREME regiment being cost-effective compared with nivolumab and ipilimumab, was 64%. Importantly, there was heterogeneity in the cost-effectiveness probabilities, based on primary sites and expression levels of PD-L1. Therefore, tailored treatment based on individual patient and clinical characteristics, remains important, and may impact the cost-effectiveness of the regimens under study.

## Introduction

The term squamous cell carcinoma of the head and neck (SCCHN) refers to tumors originating from the mucosa of the upper aerodigestive tract, which accounts for nearly 90% of all head and neck tumors^1,2^. It is common to diagnose patients with locally advanced disease, but recurrences happen in 30–45% of patients within a year^3^. There is a poor prognosis for patients with recurrent or metastatic (R/M) SCCHN. It has been traditional to treat R/M SCCHN with platinum-based chemotherapy (cisplatin or carboplatin) and 5-fluorouracil (5-FU) or a taxane (paclitaxel or docetaxel). According to the results of the trials, the median overall survival (OS) ranged from 5.0 to 8.7 months for platinum-based combination regimens^3,4^. As a result, SCCHN has limited treatment options.

Head and neck cancer treatment has seen tremendous success with immune therapy using checkpoint inhibitors. In the treatment of R/M SCCHN, the US Food and Drug Administration and the European Medicines Agency have approved the anti-PD-1 antibodies pembrolizumab and nivolumab^5–8^. In addition to PD-1, SCCHN may also evade antitumor immunity through multiple mechanisms, including programmed death-ligand 1 (PD-L1) and cytotoxic T-lymphocyte-associated antigen 4 (CTLA-4)^9^. PD-L1 expression on tumor cells and tumor-associated immune cells (measured as a combined positive score [CPS]) is a potential biomarker to identify patients likely to respond to immune checkpoint inhibitors. Currently, ICIs have shown efficacy in a variety of cancers, but the threshold for CPS differs for different cancers. There still remains an unmet need to improve clinical outcomes in R/M SCCHN despite advancements in treatment. Nivolumab and ipilimumab were recently introduced as the first-line treatment in R/M SCCHN in comparison with EXTREME regimen (cetuximab + cisplatin/carboplatin + fluorouracil) in the CheckMate 651 trial^10^. All randomly assigned or PD-L1 CPS ≥ 20 populations in this pivotal trial did not experience a significant OS benefit in favor of first-line nivolumab plus ipilimumab over EXTREME. A median OS of 15.7 months was seen in patients with CPS ≥ 1 (nivolumab plus ipilimumab arm) versus 13.2 months in EXTREME arm, with an HR of 0.82 (95% CI, 0.69–0.97). In addition, 28.2% of patients receiving nivolumab plus ipilimumab had grade ≥ 3 treatment-related adverse events (AEs) compared to 70.7% of those getting EXTREME regimen.

However, immunotherapy has a higher medical expense compared to traditional chemotherapy, despite this impressive clinical outcome. An economic evaluation of the new treatments is urgently needed because cost-effectiveness analyses are useful for clinicians and decision makers when allocating limited health care resources. In SCCHN, cost-effectiveness studies have been conducted on other immune checkpoint inhibitors (ICIs), such as pembrolizumab^1^, but it is not clear whether dual ICI-based regimens will be economically viable and which populations will benefit most from such treatments. On the other hand, the main rationale for conducting a cost-effectiveness analysis of nivolumab plus ipilimumab compared with EXTREME regimen is that the differences in toxicity. Furthermore, the  hazard ratio (HR) had a better trend for OS, especially for PD-L1 CPS ≥ 1 population with significant difference, and the different subgroup population showed different clinical efficacy. Nivolumab plus ipilimumab or the EXTREME regimen were evaluated for cost-effectiveness, as a first-line treatment for R/M SCCHN, in the United States, by all randomly assigned patients, as well as, patients with PD-L1 high expression, based on the results of CheckMate 651.

## Methods

### Patients and intervention

CheckMate 651 was the basis of this economic evaluation study^10^. There were no real human participants in this study, so the institutional review board was not required to approve it. This study was prepared in accordance with the CHEERS 2022 (Consolidated Health Economic Evaluation Reporting Standards 2022) reporting^11^. A cohort of patients included in the CheckMate 651 trial was used as the target patient population.

According to CheckMate 651, inclusion criteria required participants to be at least 18 years old; participants had to have a pathologically confirmed R/M SCCHN, and without prior systemic therapy; had documented tumor PD-L1 and HPV status; had an Eastern Cooperative Oncology Group (ECOG) performance status score of 0 or 1^10^.

In the nivolumab plus ipilimumab group, nivolumab (3 mg/kg, once every 2 weeks) and ipilimumab (1 mg/kg, once every 6 weeks) was administered until disease progression, intolerable toxicity, or > 2 years. Participants in the EXTREME group received cetuximab (400 mg/m^2^ loading dose, then 250 mg/m^2^ per week), and carboplatin (area under the curve 5 mg/m^2^) or cisplatin (100 mg/m^2^) and 5-fluorouracil (1000 mg/m^2^ per day for 4 consecutive days) every 3 weeks for six cycles. Furthermore, the maintenance dosage of cetuximab was 250 mg/m^2^. Investigator decided the choice of cisplatin or carboplatin. All drugs were administered intravenously^10^.

### Partitioned survival model

Patients with R/M SCCHN were compared using a partitioned survival model (PSM) to compare cost and effectiveness between the two competing regimens (nivolumab plus ipilimumab versus EXTREME) from the third-party payer perspective in the United States. In the model, there were three states of health: progression-free survival (PFS), progressed disease (PD), and death. All patients were initially assigned a PFS state, which could be maintained or redistributed throughout each cycle^12^.

An estimated PFS percentage is based on the area under the curve (AUC) for the PFS, whereas a death percentage is obtained by subtracting the OS curve from 1. As a result of the AUC between the PFS and OS curves, the PD state was defined. Considering the frequency of nivolumab plus ipilimumab and EXTREME, parameter calculation was facilitated by setting the model cycle length to 1 week. The time horizon was 15 years given that more than 98% of the cohort died. Parameters performed to identify the sensitive factors are shown in Table 1.

**Table 1:** Key model inputs.

Parameter	Value (95% CI)	Distribution	Source
Log-logistic OS survival model of nivolumab plus ipilimumab^a^	μ = 1.2311, σ = 0.0169	Log-logistic	^10^
Log-logistic PFS survival model of nivolumab plus ipilimumab^a^	μ = 1.2612, σ = 0.0594	Log-logistic	^10^
Log-logistic OS survival model of EXTREME^a^	μ = 1.5669, σ = 0.0165	Log-logistic	^10^
Log-logistic PFS survival model of EXTREME^a^	μ = 1.8949, σ = 0.0337	Log-logistic	^10^
Drug costs per 1 mg, $			
Nivolumab	30 (29–32)	Gamma	^14^
Ipilimumab	166 (157–174)	Gamma	^14^
Cisplatin	0.17 (0.14–0.2)	Gamma	^14^
Carboplatin	0.05 (0.04–0.06)	Gamma	^14^
5-Fluorouracil	0.005 (0.004–0.006)	Gamma	^14^
Cetuximab	7.04 (5.63–8.44)	Gamma	^14^
Cost of terminal care per patient^b^	11,126 (8901–13,351)	Gamma	^15^
Best supportive care cost per cycle	4645 (3716–5574)	Gamma	
Drug administration cost, $			
First hour	150.34 (123.55–198.06)	Gamma	^16^
Additional hour	32.23 (27.29–41.02)	Gamma	^16^
Cost of managing AEs (grade ≥ 3), $^c^			
Nivolumab plus ipilimumab	893 (848–939)	Gamma	^17,18^
EXTREME	9692 (9207–10,196)	Gamma	^17,18^
Immunohistochemical test	76.45 (35.57–146.77)	Gamma	^16^
Follow-up cost per cycle	380 (304–456)	Gamma	^2^
Health utilities			
Disease status utility per y			
Utility PFS	0.805 (0.644–0.966)	Beta	^19^
Utility PD	0.749 (0.599–0.899)	Beta	^19^
Death	0	NA	
Drug toxic effects disutility^c^			
Nivolumab plus ipilimumab	0.016 (0.013–0.019)	Beta	^17,20,21^
EXTREME	0.152 (0.121–0.182)	Beta	^17,20,21^
Other inputs			
CT scans of the head and neck (per time)	84.23 (41.99–160.29)	Gamma	^16^
Body surface area, m^2^	1.86 (1.40–2.23)	Normal	^2^
Body weight, kg	70 (50–91)	Normal	^2^

*EXTREME* cetuximab plus cisplatin/carboplatin plus fluorouracil, *PFS* progression-free survival, *OS* overall survival, *HR* hazard ratio, *PD* progressed disease, *AEs* adverse events.

^a^Only expected values are presented for these survival model parameters.

^b^Overall total cost per patient regardless of treatment duration.

^c^The mean cost and utility toll of adverse events weighted by the frequency of occurrence.

### Clinical data inputs

Data from the CheckMate 651 trial^10^ were used to determine clinical efficacy and safety. To extract PFS and OS data points from the relevant Kaplan–Meier (K–M) survival curves, the GetData Graph Digitizer (version 2.26)^13^ was used in the absence of individual patient data (IPD). For extrapolating survival curves beyond the clinical trial follow-up period, different parametric distributions were fitted, including Log-logistic, Exponential, Gamma, Weibull, Lognormal, Gompertz, and Generalized gamma distributions.

In addition to graphical validation, Bayesian information criterion (BIC) and Akaike information criterion (AIC) were used to assess the distribution with the best fit (Supplementary Table 1). R version 4.0.5 was used to calculate the AIC and BIC. Supplementary Fig. 1 compares the model-fitted and original K–M curves. A summary of the key clinical input data is provided in Table 1^2,10,14–21^.

### Costs

Costs associated with drugs, laboratory tests, managing AEs, terminal care, and providing best supportive care were considered (Table 1). Terminal care is end of life care, in this study, overall total cost per patient regardless of treatment duration. End of life care should help you to live as well as possible until you die and to die with dignity. Best supportive care as proactive, reliable, and valued care. Detailed drug Average Sales Price (ASP) were gathered from the Center for Medicare and Medicaid Services (CMS)^14^. In Table 1, all costs have been inflated to 2022 US dollars using Tom's Inflation Calculator^22^. Body weight and body surface area were provided in CheckMate 651, and the dosage of nivolumab and ipilimumab is calculated by body weight, and the dosage of EXTREME is calculated by body surface area, hence, we assumed that the average body surface area (BSA) and body weight were assumed to be 1.86 m^2^ and 70 kg, respectively^2^. AEs in grade ≥ 3 were included in the model. We have also evaluated the management costs of grade ≥ 3 AEs, the costs related to management of grade ≥ 3 AEs were obtained from the literature (Supplementary Table 2)^17,18^.

### Utilities

The PSM assigned utility values to each health state anchored in 0 (death) and 1 (perfect health). We derived utility values from the published literature since CheckMate 651 did not report such data. As a whole, SCCHN was associated with PD health utilities of 0.749 and PFS health utilities of 0.805^19^. The disutility values caused by grade ≥ 3 AEs were also considered in accordance with the relevant literature (Supplementary Table 2)^20,21^.

### Base-case analysis

Comparisons were made between treatment groups based on overall costs, life years, quality-adjusted life years (QALYs), incremental cost-effectiveness ratios (ICERs), incremental net monetary benefit (INMB), and incremental net health benefit (INHB). INMB measures the difference in net monetary benefit between a standard, a positive incremental net monetary benefit indicating that the intervention is cost-effective compared with the standard at the given willingness to pay (WTP). INHB defined as the difference in mean effectiveness of a new treatment compared with a standard, adjusted for cost difference by subtracting the health foregone if purchasing care at the rate of a marginally cost-effective therapy. In this study, a threshold of $150,000 was established for WTP for QALYs^12^. A lower ICER than the WTP threshold implies cost-effectiveness, according to the recommendation^23^. Based on a 3% discount rate per year, the cost and effectiveness of the project were evaluated^24^.

### Sensitivity analysis

Using one-way sensitivity analysis for input parameter values, we evaluated the robustness of the model and identified variables that have substantial impacts on analysis results. One-way sensitivity analysis involves adjusting each input parameter to its minimum and maximum values one by one, using a 95% confidence interval or a change of ± 20% from the base case reported in the literature.

Probabilistic sensitivity analysis (PSA) is a technique used in economic modelling that allows the modeller to quantify the level of confidence in the output of the analysis, in relation to uncertainty in the model inputs. By sampling all input parameters from the pre-specified distributions simultaneously, 10,000 Monte Carlo simulations were performed for PSA. Gamma distribution was used to sample all costs. Beta distribution was used to sample utility values and probabilities. All parameters in the Table 1 are included in the PSA. The probabilities of cost-effectiveness at various WTP thresholds were illustrated using cost-effectiveness acceptability curves (CEACs) based on the results of 10,000 iterations.

### Subgroup analyses

Analysis of subgroups of patients was performed to explore the impact of different patient characteristics on the outcomes. By varying the HR for OS for the different subgroups derived from CheckMate 651^10^, including age, primary tumor site, smoking status, and tumor PD-L1 expression, subgroup analyses were constructed for each group. The statistics in this study were performed using R 4.0.5 and the hesim and heemod packages.

## Results

### Base case analysis

Compared patients with R/M SCCHN to those receiving EXTREME, nivolumab plus ipilimumab decreased effectiveness by 0.076 QALYs and 0.154 overall life-years, while reducing additional cost of $572. Hence, EXTREME was related to ICER of $7545/QALY compared with nivolumab plus ipilimumab. Additionally, EXTREME had an INMB of $113,267 and an INHB of 0.076 QALYs at a WTP of $150,000/QALY versus nivolumab plus ipilimumab (Table 2).

**Table 2:** Summary of cost and outcome results in the base-case analysis of overall patients.

Factor	EXTREME	Nivolumab plus ipilimumab	Incremental change^a^
Cost, $			
Drug	290,970	322,595	− 31,625
Nondrug^b^	565,153	532,956	32,197
Overall	856,123	855,551	572
Life-years			
Progression-free	0.466	0.847	− 0.381
Overall	2.534	2.380	0.154
QALYs	1.747	1.671	0.076
ICERs, $			
Per life-year	NA	NA	3711
Per QALY	NA	NA	7545
INHB, QALY, at threshold 150,000^c^	NA	NA	0.076
INMB, $, at threshold 150,000^c^	NA	NA	113,267

*EXTREME* cetuximab plus fluorouracil plus carboplatin/cisplatin, *ICER* incremental cost-effectiveness ratio, *INHB* incremental net health benefit, *INMB* incremental net monetary benefit, *NA* not applicable, *QALYs* quality-adjusted life years.

^a^Change in cost and change in QALYs represent the results of EXTREME minus nivolumab plus ipilimumab.

^b^Nondrug cost includes the costs of adverse event management, subsequent best supportive care per patient, and follow-up care covering physician monitors, drug administration, and terminal care.

^c^INHB and INMB mean EXTREME compared with nivolumab plus ipilimumab.

### Sensitivity analysis

**Figure 1:**
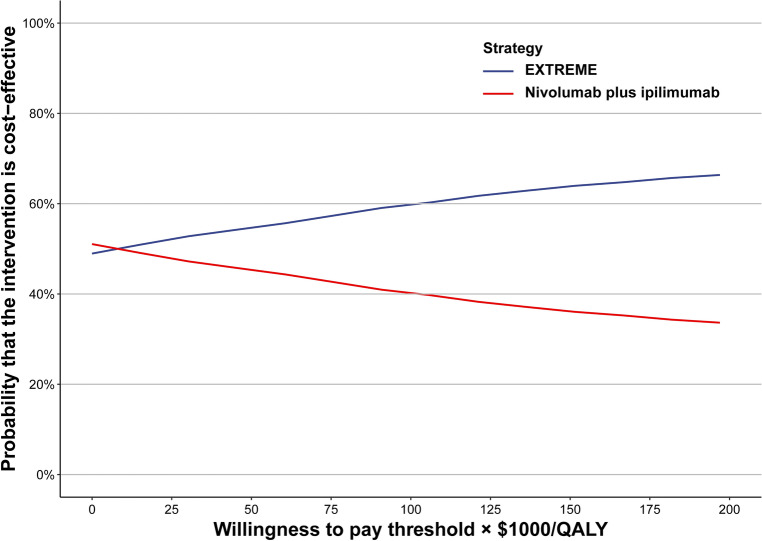
Acceptability curves of cost-effectiveness for nivolumab plus ipilimumab and EXTREME. *QALY* quality-adjusted life year, *EXTREME* cisplatin/carboplatin + cetuximab + fluorouracil.

As shown in Fig. 1, the CEAC displayed the PSA results. With an increase in WTP thresholds, EXTREME became increasingly cost-effective. Results of PSA are shown in Supplementary Fig. 2. According to the scatter plot, EXTREME had a 63.86% probability of being considered cost-effective compared with nivolumab plus ipilimumab (36.14%) at a WTP threshold of $150,000/QALY for the total population.

Supplementary Fig. 3 presents a tornado plot indicating the one-way sensitivity analysis for EXTREME compared to nivolumab plus ipilimumab. Variations in HR for OS and body weight were generally the strongest influences on ICERs. Only weak or moderate relationships were found in the remaining parameters, such as the cost of drugs (cetuximab, nivolumab, and ipilimumab) and health state utility values. Additionally, we evaluated the relevance of body weight with regard to the ICER when comparing nivolumab plus ipilimumab with EXTREME. Nivolumab plus ipilimumab could be considered as a cost-effective option when the weight of patient was less than 63.19 kg at WTP threshold of $150,000/QALY (Supplementary Fig. 4).

### Subgroup analysis

A greater probability of nivolumab plus ipilimumab being cost-effective was observed in subgroups with superior survival. According to primary sites and expression levels of PD-L1, there were heterogeneous cost-effectiveness probabilities for nivolumab plus ipilimumab. Nivolumab plus ipilimumab had > 99% probability of being considered cost-effective in the hypopharynx primary site and PD-L1 high expression (CPS ≥ 1, 1–19, or ≥ 20) subgroups, at a WTP threshold of $150,000/QALY. It is unlikely that nivolumab plus ipilimumab would be considered cost-effective in patients with PD-L1 CPS < 1 (Table 3).

**Table 3:** Summary of subgroup analyses obtained by varying the hazard ratios (HRs) for overall survival.

Subgroup	Unstratified hazard ratio^a^	Change in cost, $^b^	Change in QALYs^b^	ICER, $/QALY^b^	WTP of $150,000/QALY	
					Cost-effectiveness probability of nivolumab plus ipilimumab, %	INHB^b^
Age (years)						
< 65	0.88	9781	0.230	42,454	18.67	0.165
65–75	0.99	− 36,926	− 0.004	9,201,654	4.87	0.242
≥ 75	1.37	− 290,739	− 0.542	535,959	0	1.396
Sex						
Male	0.95	739	0.076	9743	27.23	0.071
Female	0.91	41,216	0.162	254,793	67.41	− 0.113
ECOG PS						
0	0.83	131,614	0.354	372,277	99.63	− 0.524
1	0.97	− 18,430	0.035	− 523,213	13.39	0.158
Primary site						
Oral cavity	0.94	8602	0.093	92,924	34.19	0.035
Oropharynx	0.93	20,611	0.118	174,599	46.19	− 0.019
Hypopharynx	0.84	119,570	0.328	364,557	99.05	− 0.469
Larynx	1.02	− 63,467	− 0.060	1,052,195	0.91	0.363
Smoking status						
Current or former smoker	0.91	41,216	0.162	254,793	66.51	− 0.113
Never smoker	1.13	− 149,707	− 0.243	615,388	0	0.755
p16 status						
OPC p16-positive	1.19	− 190,342	− 0.329	577,709	0	0.939
OPC p16-negative or non-OPC	0.89	62,585	0.207	302,202	83.10	− 0.210
Prior chemotherapy						
Yes	0.87	84,750	0.254	333,507	93.56	− 0.311
No	1.00	− 45,930	− 0.023	1,987,036	3.07	0.283
Disease status at study entry						
Locally recurrent	1.00	− 54,776	− 0.042	1,307,903	1.45	0.323
Locally recurrent and metastatic	0.93	20,611	0.118	174,599	46.35	− 0.019
Metastatic	0.87	84,750	0.254	333,507	93.74	− 0.311
Tumor PD-L1 expression						
< 1% and nonevaluable	1.18	− 183,851	− 0.316	582,347	0	0.910
≥ 1%	0.80	169,124	0.433	390,485	99.98	− 0.694
PD-L1 CPS						
< 1	1.66	− 403,769	− 0.782	516,163	0	1.910
≥ 1	0.81	156,386	0.406	385,101	99.91	− 0.636
1–19	0.83	131,614	0.354	372,277	99.56	− 0.524
≥ 20	0.81	155,126	0.403	384,530	99.91	− 0.631

*CPS* combined positive score, *ECOG PS* Eastern Cooperative Oncology Group performance status, *EXTREME* cetuximab plus cisplatin/carboplatin plus fluorouracil, *HR* hazard ratio, *ICER* incremental cost-effectiveness ratio, *OPC* oropharyngeal cancer, *OS* overall survival, *PD-L1* programmed death-ligand 1, *QALY* quality-adjusted life-year, *WTP* willingness to pay.

^a^HR for OS represents the HR of nivolumab plus ipilimumab versus EXTREME for OS.

^b^INHB represents the results of EXTREME minus nivolumab plus ipilimumab.

## Discussion

This study compares the cost-effectiveness of the nivolumab plus ipilimumab regimen compared with EXTREME, in R/M SCCHN patients, from the standpoint of third-party payers in the United States. Despite not meeting its primary end point of OS in all randomly assigned populations, nivolumab plus ipilimumab had a better safety profile than EXTREME in the CheckMate 651 study. An economic evaluation provides an organized method for analyzing the available alternatives and determining the effects on health, health care costs, and other valuable effects that should be considered when determining how to use clinical evidence effectively.

With the current price of EXTREME in the United States and the WTP threshold of $150,000, our base-case analysis shows that it is cost-effective, resulting in an improvement in QALYs of 0.076 and overall life-years of 0.154, and an additional $572 cost compared to patients receiving nivolumab plus ipilimumab, and an ICER of $7545/QALY. The results of one-way sensitivity analyses showed that variation in HR for OS and body weight were the most important factors influencing the results of base-case analyses. Various people will use different number of administrations and/or amounts of nivolumab and ipilimumab per cycle due to the use of nivolumab and ipilimumab based on body weight, which results in significant differences in costs. The results of subgroup analysis indicated a higher probability of cost-effectiveness in subgroups with better survival advantages than in subgroups with poor survival advantages. A more personalized approach to treatment based on individual factors could lead to better outcomes economically. The results of this model are robust, based on the results of a comprehensive one-way sensitivity analysis and a PSA. Using the WTP threshold of $150,000/QALY to determine cost-effectiveness, EXTREME had a probability of 63.86%.

Often, ICIs are expensive because they require a lot of research and development^25,26^. As a result, ICIs are typically not cost-effective as they are mentioned in the literature^27^. Patients with recurrent SCCHN benefited from nivolumab, the first immunotherapy to produce clinical benefits. Based on the results of the CheckMate 141 trial, some cost-effectiveness analyses evaluated nivolumab as a second-line treatment for R/M SCCHN^19,28–30^. Studies conducted by Ward and Haddad in the United States found that nivolumab was cost-effective as a second-line treatment for R/M SCCHN patients at a threshold of $150,000/QALY^19,28^. However, according to previously published studies, nivolumab was not cost-effective in Switzerland and Canada, although the studies also indicated that the price of nivolumab could be lowered to make it cost-effective^29,30^. The results of two additional cost-effectiveness analyses for nivolumab did not show a correlation between higher tumor PD-L1 expression and increased cost-effectiveness^28,29^. Those two studies indicated that factors likely to positively impact the cost-effectiveness of nivolumab include better baseline quality-of-life, poor tolerability of standard treatments and/or a lower cost of nivolumab.

As a second-line treatment for R/M SCCHN, pembrolizumab monotherapy has been shown to be cost-effective in China and the United States in previously published studies^31^. For R/M SCCHN patients in the United States, a cost-effectiveness assessment was performed with pembrolizumab monotherapy and pembrolizumab plus chemotherapy. This study found that pembrolizumab monotherapy and pembrolizumab plus chemotherapy were cost-effective compared with the EXTREME regimen^32^. Pembrolizumab alone appeared to be optimal for the general population and for patients with CPS ≥ 1 in another study from the payer's perspective in China, when it came to patients with CPS ≥ 20, immunotherapy did not outperform EXTREME either with or without chemotherapy^33^.

Previous research has targeted on the economic evaluations of ICIs as a primary treatment for SCCHN. The differences between the results of their study and ours may be explained as follows. First, the initial health utility was unclear, the disutilities associated with grades 3–5 AEs were not described, which largely contributed to the inconsistent results between our studies. Second, no costs for subsequent treatment, terminal care, and best supportive care were considered. Furthermore, body weight is one of the most important factors influencing the results of base-case analyses. For US patients with platinum-refractory SCCHN, it may not be wise to completely abandon weight-based dosing in favor of a fixed dose, and dosing regimens should be individualized. We recommend taking into consideration both the WTP threshold and patient weight to make an optimal clinical decision. Based on the results of our study, nivolumab plus ipilimumab could be considered as a cost-effective option when the weight of patient was less than 63.19 kg at WTP threshold of $150,000/QALY.

It is important to note that the study has several limitations. First, we omitted other ICIs, including durvalumab and pembrolizumab, that have been evaluated for the treatment of R/M SCCHN due to the lack of head-to-head data. Additionally, the initial clinical trial may have had some biases, resulting in our study to be influenced by it. It may have been biased to calculate subsequent treatment costs based on the proportions of subsequent treatments reported as a single agent. This study would benefit from additional clinical data. Second, due to the lack of reliable and accurate quality of life information in the CheckMate 651 trial, the utilities for SCCHN patients in the model were not derived from it. The QALYs reported in our study may have been impacted by having to use utility data reported in the literature, as it was not reported in the original clinical publication. It is worth mentioning that ICERs do not reach the acceptable threshold when utility value fluctuations occur, according to our one-way sensitivity analysis. Third, there are also limitations to this study related to grades 1–2 AEs. Due to the higher risk of grades 1–2 AEs of nivolumab plus ipilimumab group (44.02%) compared with EXTREME (26.76%), reported by CheckMate 651. The immune-related AEs and grades 1–2 AEs were not considered, which could have resulted in an overestimation of the outcomes associated with nivolumab plus ipilimumab. This limitation, however, had negligible significance in outcomes according to sensitivity analysis. It is important to note that general clinical practice cannot ignore AEs. Fourth, the present analysis examined the economic outcomes of subgroups prespecified by the CheckMate 651 trial. Economic information for the subgroups may help tailor treatment decisions of physicians, patients, and policy makers. Further work needs to confirm who may or may not benefit from treatment with nivolumab plus ipilimumab. Furthermore, due to the most of CI for the HR either crossed or included, and the results needs to be interpreted very carefully. Additionally, a subgroup analysis of PFS data was not available, which reduced the robustness of our findings. Last, the reported K–M survival curves of OS and PFS data were fitted using parametric distributions in order to account for health outcomes that occurred beyond the follow-up period of the CheckMate 651 study. This may have led to uncertainty in the predictions of the model. The findings of the sensitivity analysis show that this conclusion is typically robust, suggesting that this constraint may not be a significant factor.

## Conclusion

The EXTREME regimen is cost-effective compared with nivolumab plus ipilimumab for all randomly assigned populations with R/M SCCHN at a WTP threshold of $150,000/QALY from the payer's perspective in the United States. Given a WTP threshold of $150,000 per QALY, the probability of the EXTREME regiment being cost-effective compared with nivolumab and ipilimumab, was 64%. Importantly, there was heterogeneity in the cost-effectiveness probabilities, based on primary sites and expression levels of PD-L1. Therefore, tailored treatment based on individual patient and clinical characteristics, remains important, and may impact the cost-effectiveness of the regimens under study.

### Supplementary Information

Supplementary Information.

## Data Availability

The datasets generated during and/or analyzed during the current study are available from the corresponding author on reasonable request.
