# Supplementary material for: Nivolumab plus ipilimumab versus the EXTREME regimen in recurrent/metastatic squamous cell carcinoma of the head and neck: a cost-effectiveness analysis
Source: Sci Rep. 2024 Mar 21;14:6807. doi: 10.1038/s41598-024-57277-7 (PMC10957979; doi:10.1038/s41598-024-57277-7)
Supplement: Supplementary file 1 — Supplementary Information. [file 41598_2024_57277_MOESM1_ESM.docx]

**Supplementary Content**

**Supplementary Figure 1.** Model Fitting Analysis

**Supplementary Figure 2.** Results of Probabilistic Sensitivity Analysis Showing Incremental Cost-effectiveness of Nivolumab Plus Ipilimumab Versus EXTREME.

**Supplementary Figure 3.** Tornado Diagram of One-Way Sensitivity Analyses

**Supplementary Figure 4.** Impacts of Key Factors on Incremental Cost-effectiveness Ratio

**Supplementary Table 1.** Akaike Information Criterion and Bayesian Information Criterion Values from Each Survival Model

**Supplementary Table 2.** Associated Costs and Disutility of Treatment-Related Adverse Events

**Supplementary Figure 1.** Model Fitting Analysis

To obtain the best model fit, the following investigations were carried out using nivolumab plus ipilimumab or EXTREME as the model fit baseline, respectively. Based on values of AIC and BIC (Supplementary Table 1), log-logistic was used to fit the OS and PFS curves of the two treatment arms. EXTREME, cetuximab, fluorouracil, and either carboplatin or cisplatin.

(A) Model-fitted versus original K-M curves for nivolumab plus ipilimumab.


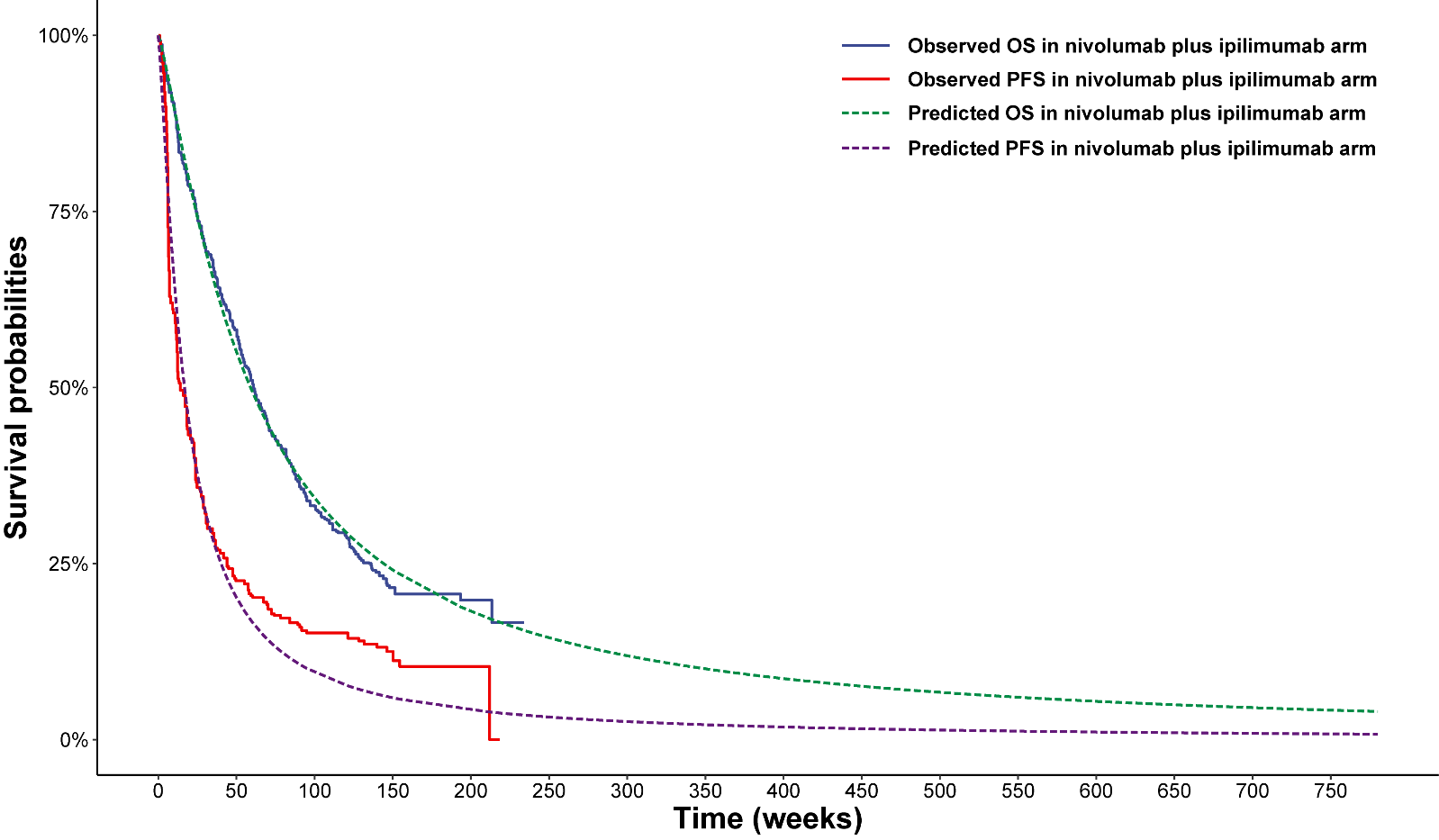


(B) Model-fitted versus original K-M curves for EXTREME.


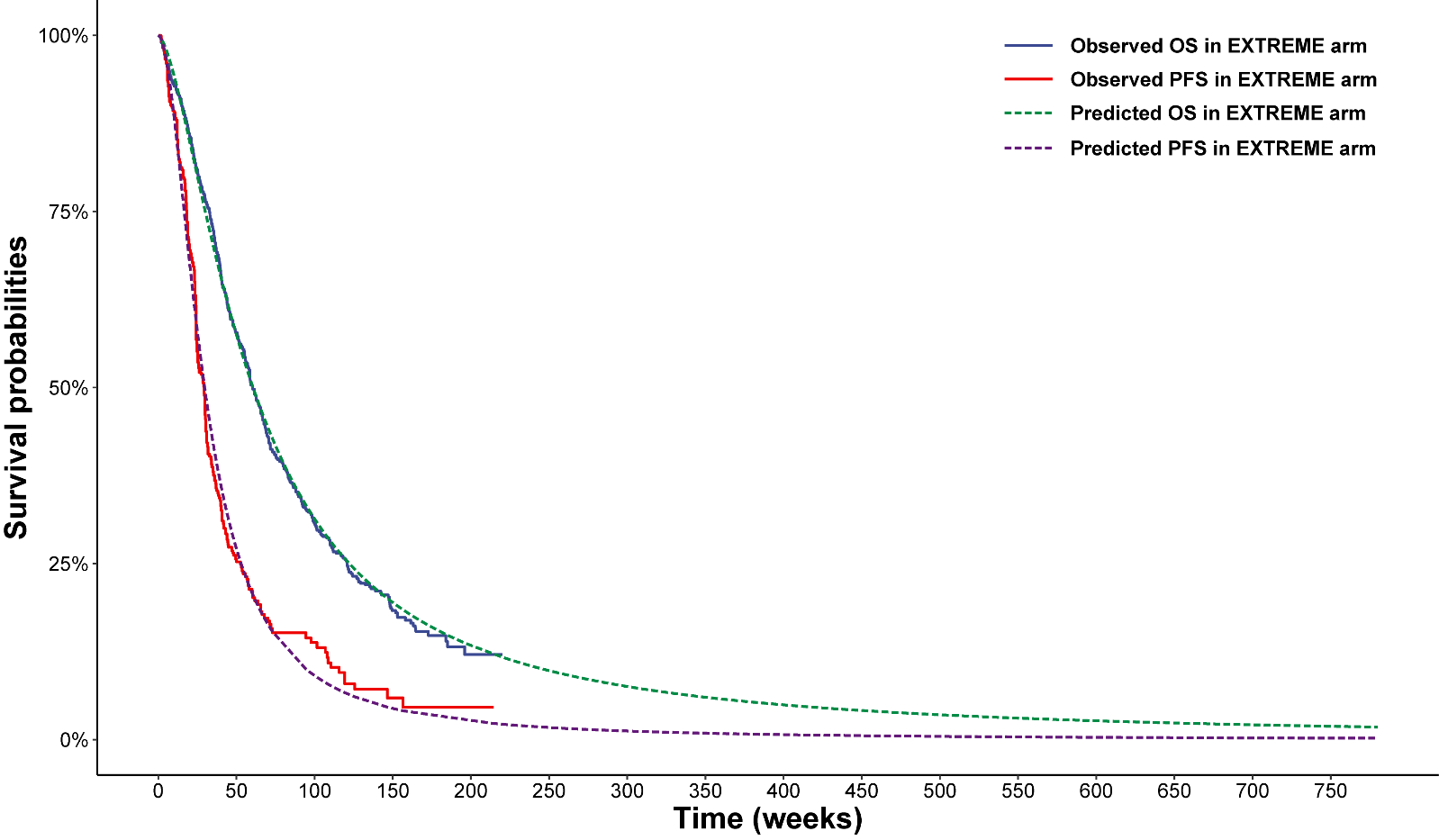


**Supplementary Figure 2.** Results of Probabilistic Sensitivity Analysis Showing Incremental Cost-effectiveness of Nivolumab Plus Ipilimumab Versus EXTREME.


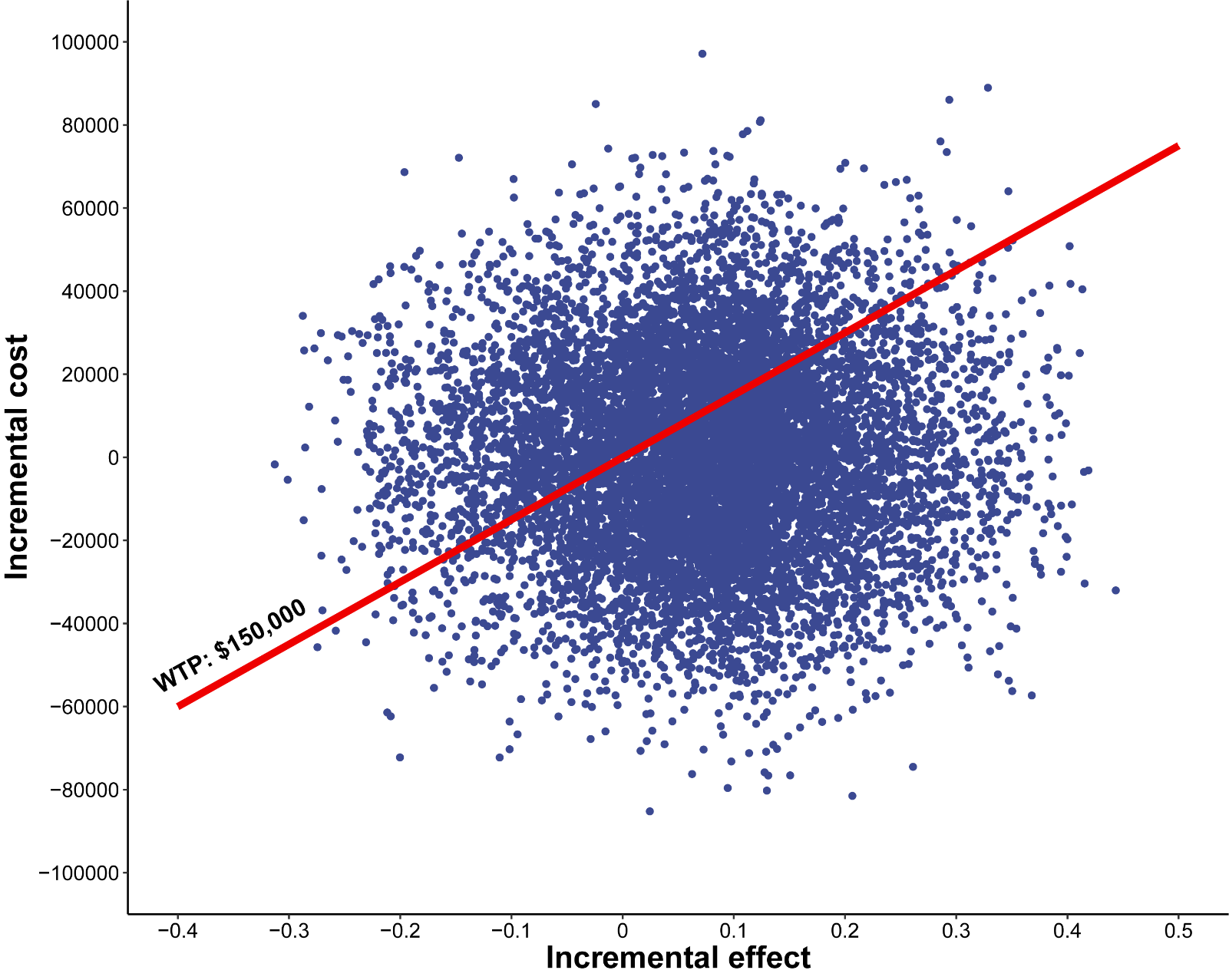


**Supplementary Figure 3.** Tornado Diagram of One-Way Sensitivity Analyses.


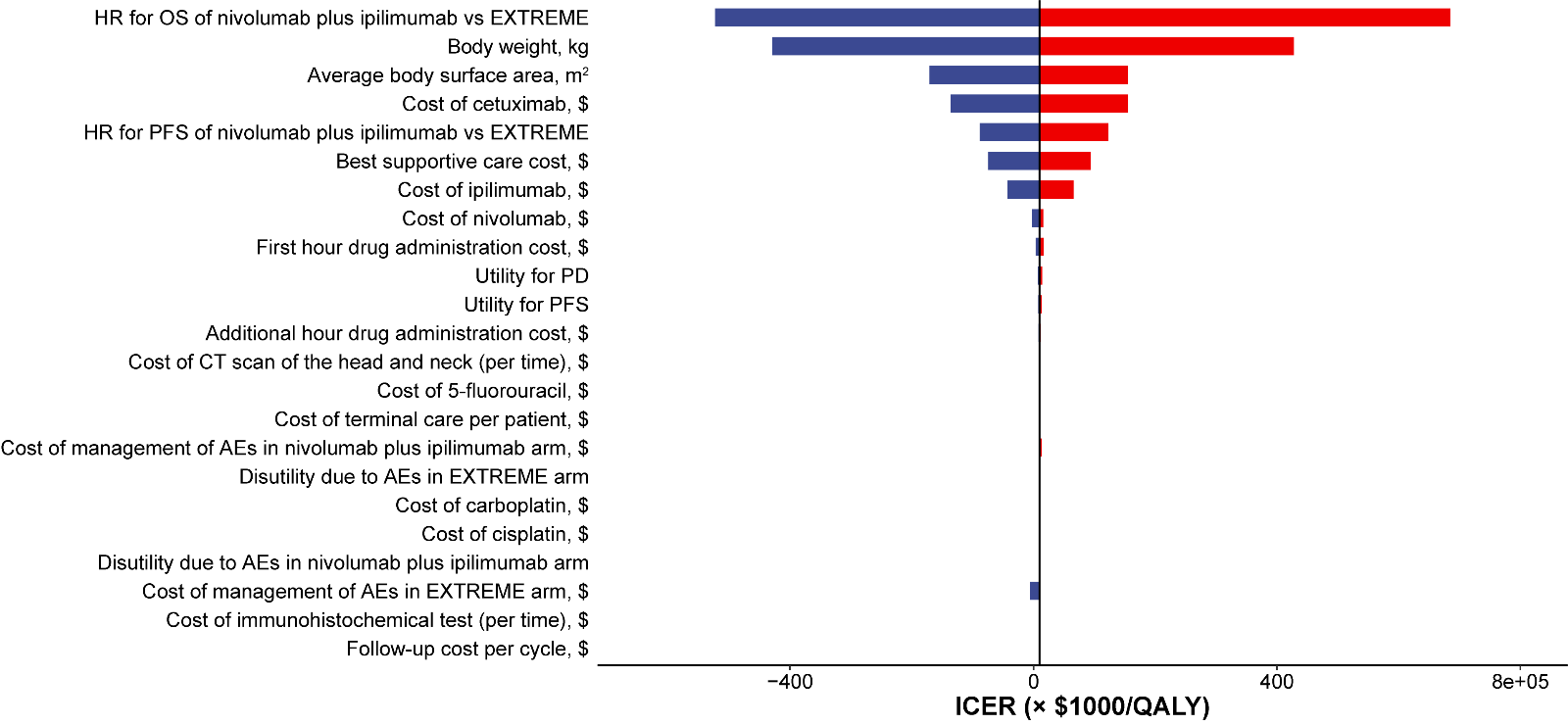


EXTREME, cetuximab, fluorouracil, and either carboplatin or cisplatin; ICER: Incremental cost-effectiveness ratio; OS, overall survival; HR, hazard ratio; PD, progressed disease; PFS, progression-free survival; AEs, adverse events.

**Supplementary Figure 4.** Impacts of Key Factors on Incremental Cost-effectiveness Ratio

The diagrams show the weights on the incremental cost-effectiveness ratio (nivolumab plus ipilimumab) for the treatment of recurrent/metastatic squamous cell carcinoma of the head and neck.


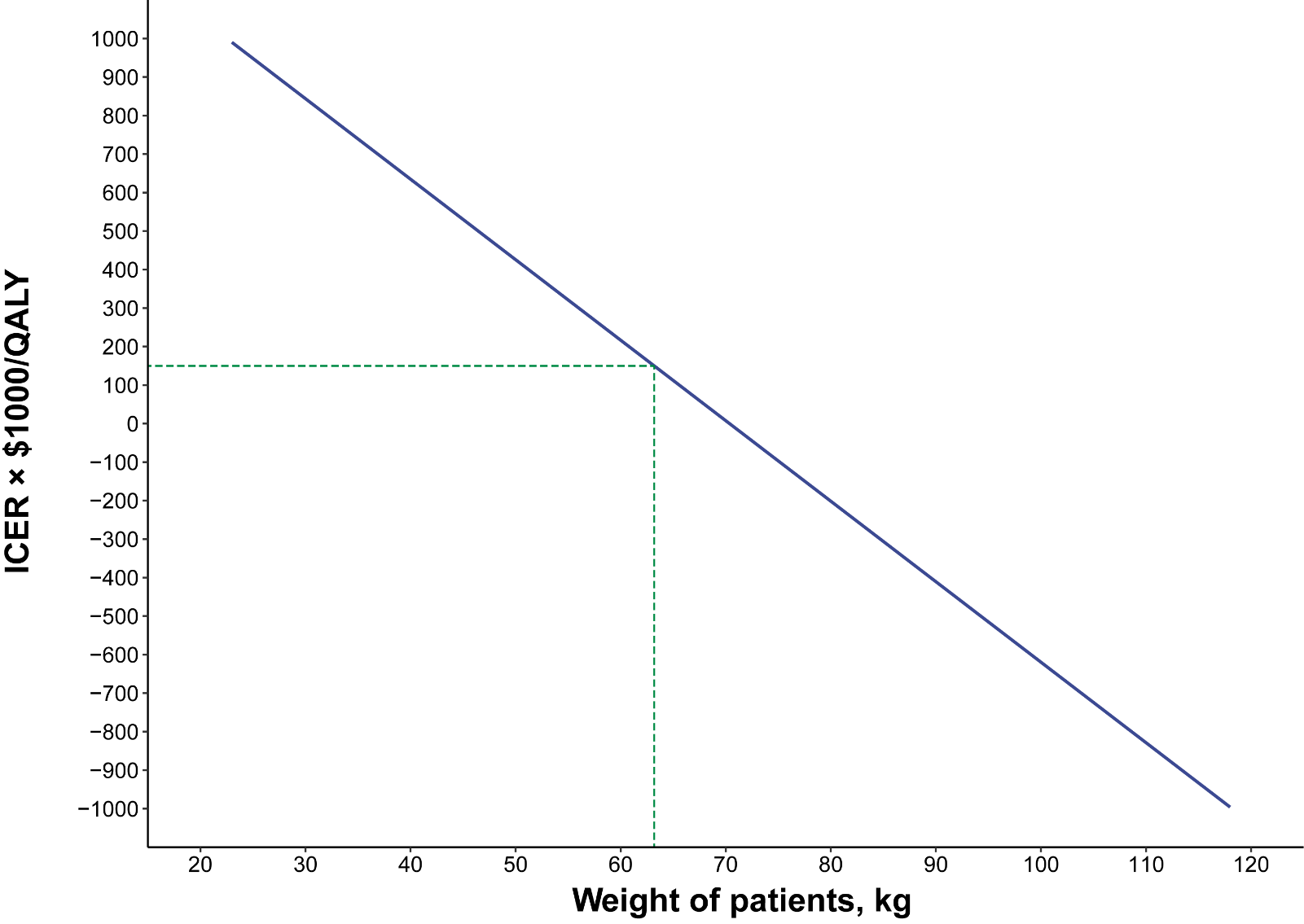


**Supplementary Table 1.** Akaike Information Criterion and Bayesian Information Criterion Values from Each Survival Model. AIC, Akaike information criterion; BIC, Bayesian Information Criterion; OS, overall survival; PFS, progression-free survival.

| **Strategies** | **Distributions** | **Parameters** | **est** | **se** | **L95%** | **U95%** | **AIC** | **BIC** |
| --- | --- | --- | --- | --- | --- | --- | --- | --- |
| **Results of OS** | | | | | | | | |
| **Nivolumab plus ipilimumab** | Exponential | rate | 0.0103 | 0.0005 | 0.0093 | 0.0114 | 3951.228 | 3955.385 |
|  | Weibull | shape | 0.9192 | 0.0412 | 0.8420 | 1.0036 | 3949.558 | 3957.872 |
|  |  | scale | 0.0149 | 0.0029 | 0.0101 | 0.0219 |  |  |
|  | Gamma | shape | 0.9154 | 0.0587 | 0.8073 | 1.0380 | 3951.285 | 3959.599 |
|  |  | rate | 0.0092 | 0.0009 | 0.0076 | 0.0111 |  |  |
|  | Lognormal | meanlog | 4.0519 | 0.0703 | 3.9142 | 4.1896 | 3954.443 | 3962.757 |
|  |  | sdlog | 1.4507 | 0.0571 | 1.3431 | 1.5670 |  |  |
|  | Gompertz | shape | -0.0041 | 0.0012 | -0.0065 | -0.0017 | 3941.174 | 3949.488 |
|  |  | rate | 0.0129 | 0.0011 | 0.0110 | 0.0152 |  |  |
|  | Log-logistic | shape | 1.2311 | 0.0550 | 1.1279 | 1.3437 | **3940.649** | **3948.963** |
|  |  | scale | 59.0927 | 3.8899 | 51.9400 | 67.2305 |  |  |
|  | Generalized gamma | mu | 4.3463 | 0.1024 | 4.1456 | 4.5471 | 3943.709 | 3956.18 |
|  |  | sigma | 1.2580 | 0.0774 | 1.1152 | 1.4193 |  |  |
|  |  | Q | 0.5339 | 0.1550 | 0.2302 | 0.8376 |  |  |
| **EXTREME** | Exponential | rate | 0.0109 | 0.0006 | 0.0099 | 0.0121 | 4126.891 | 4131.055 |
|  | Weibull | shape | 1.1274 | 0.0479 | 1.0373 | 1.2254 | 4121.356 | 4129.683 |
|  |  | scale | 0.0061 | 0.0014 | 0.0039 | 0.0095 |  |  |
|  | Gamma | shape | 1.2450 | 0.0804 | 1.0970 | 1.4129 | 4117.97 | 4126.297 |
|  |  | rate | 0.0141 | 0.0012 | 0.0119 | 0.0167 |  |  |
|  | Lognormal | meanlog | 4.0781 | 0.0548 | 3.9707 | 4.1855 | 4123.839 | 4132.166 |
|  |  | sdlog | 1.1440 | 0.0433 | 1.0623 | 1.2320 |  |  |
|  | Gompertz | shape | 0.0006 | 0.0011 | -0.0017 | 0.0028 | 4128.646 | 4136.972 |
|  |  | rate | 0.0106 | 0.0009 | 0.0090 | 0.0125 |  |  |
|  | Log-logistic | shape | 1.5669 | 0.0679 | 1.4393 | 1.7059 | **4109.391** | **4117.718** |
|  |  | scale | 60.6541 | 3.1252 | 54.8280 | 67.0993 |  |  |
|  | Generalized gamma | mu | 4.3177 | 0.0818 | 4.1573 | 4.4781 | 4113.431 | 4125.921 |
|  |  | sigma | 1.0130 | 0.0557 | 0.9094 | 1.1284 |  |  |
|  |  | Q | 0.5154 | 0.1465 | 0.2283 | 0.8025 |  |  |
| **Results of PFS** | | | | | | | | |
| **Nivolumab plus ipilimumab** | Exponential | rate | 0.0254 | 0.0013 | 0.0229 | 0.0281 | 3469.586 | 3473.743 |
|  | Weibull | shape | 0.7527 | 0.0295 | 0.6971 | 0.8128 | 3409.949 | 3418.262 |
|  |  | scale | 0.0676 | 0.0084 | 0.0530 | 0.0861 |  |  |
|  | Gamma | shape | 0.7208 | 0.0437 | 0.6401 | 0.8117 | 3439.725 | 3448.039 |
|  |  | rate | 0.0173 | 0.0017 | 0.0143 | 0.0208 |  |  |
|  | Lognormal | meanlog | 2.9232 | 0.0650 | 2.7958 | 3.0505 | 3294.447 | 3302.761 |
|  |  | sdlog | 1.3525 | 0.0513 | 1.2555 | 1.4570 |  |  |
|  | Gompertz | shape | -0.0207 | 0.0021 | -0.0248 | -0.0166 | 3319.252 | 3327.566 |
|  |  | rate | 0.0479 | 0.0032 | 0.0419 | 0.0547 |  |  |
|  | Log-logistic | shape | 1.2612 | 0.0539 | 1.1598 | 1.3715 | **3301.067** | **3309.381** |
|  |  | scale | 16.8291 | 1.1046 | 14.7976 | 19.1395 |  |  |
|  | Generalized gamma | mu | 2.2809 | 0.0950 | 2.0947 | 2.4671 | 3243.365 | 3255.836 |
|  |  | sigma | 1.1942 | 0.0507 | 1.0989 | 1.2977 |  |  |
|  |  | Q | -1.0612 | 0.1344 | -1.3246 | -0.7977 |  |  |
| **EXTREME** | Exponential | rate | 0.0217 | 0.0013 | 0.0194 | 0.0243 | 2908.99 | 2913.153 |
|  | Weibull | shape | 1.2042 | 0.0494 | 1.1112 | 1.3050 | 2892.717 | 2901.043 |
|  |  | scale | 0.0100 | 0.0020 | 0.0068 | 0.0148 |  |  |
|  | Gamma | shape | 1.5064 | 0.1031 | 1.3174 | 1.7226 | 2878.494 | 2886.821 |
|  |  | rate | 0.0357 | 0.0033 | 0.0298 | 0.0428 |  |  |
|  | Lognormal | meanlog | 3.3999 | 0.0501 | 3.3017 | 3.4981 | 2850.752 | 2859.078 |
|  |  | sdlog | 0.9590 | 0.0393 | 0.8849 | 1.0393 |  |  |
|  | Gompertz | shape | -0.0006 | 0.0018 | -0.0041 | 0.0028 | 2910.86 | 2919.186 |
|  |  | rate | 0.0221 | 0.0017 | 0.0191 | 0.0257 |  |  |
|  | Log-logistic | shape | 1.8949 | 0.0904 | 1.7257 | 2.0808 | **2839.255** | **2847.581** |
|  |  | scale | 29.6866 | 1.3787 | 27.1038 | 32.5156 |  |  |
|  | Generalized gamma | mu | 3.4456 | 0.0755 | 3.2976 | 3.5936 | 2852.118 | 2864.608 |
|  |  | sigma | 0.9430 | 0.0435 | 0.8614 | 1.0323 |  |  |
|  |  | Q | 0.1107 | 0.1380 | -0.1597 | 0.3811 |  |  |

**Supplementary Table 2.** Associated Costs and Disutility of Treatment-Related Adverse Events

| **Adverse Event^a^** | **No. of patients (%)** | **Costs in 2022 USD^c^** | **Reference** | **Disutility** | **Reference** |
| --- | --- | --- | --- | --- | --- |
| **Nivolumab plus ipilimumab^b^** |  |  |  |  |  |
| Fatigue, asthenia | 10 (2%) | 11,393 | Konidaris et al, 2020 | 0.288 | Nafees et al, 2017 |
| Rash | 8 (2%) | 6,487 | Konidaris et al, 2020 | 0.156 | Nafees et al, 2017 |
| Decreased appetite, nausea, vomiting | 1 (0) | 18,798 | Wong et al, 2018 | 0.204 | Nafees et al, 2017 |
| Anaemia | 2 (0) | 8,802 | Konidaris et al, 2020 | 0.072 | Freeman et al, 2015 |
| Diarrhoea | 8 (2%) | 18,366 | Wong et al, 2018 | 0.216 | Nafees et al, 2017 |
| Neutropenia | 3 (1%) | 20,276 | Wong et al, 2018 | 0.348 | Nafees et al, 2017 |
| Decreased platelet count | 1 (0) | 7,789 | Konidaris et al, 2020 | 0.108 | Konidaris et al, 2021 |
| Weighted averaged |  | 893 |  | 0.016 |  |
| **EXTREME^b^** |  |  |  |  |  |
| Fatigue, asthenia | 21 (5%) | 11,393 | Konidaris et al, 2020 | 0.288 | Nafees et al, 2017 |
| Rash | 28 (6%) | 6,487 | Konidaris et al, 2020 | 0.156 | Nafees et al, 2017 |
| Decreased appetite, nausea, vomiting | 25 (6%) | 18,798 | Wong et al, 2018 | 0.204 | Nafees et al, 2017 |
| Anaemia | 54 (12%) | 8,802 | Konidaris et al, 2020 | 0.072 | Freeman et al, 2015 |
| Diarrhoea | 10 (2%) | 18,366 | Wong et al, 2018 | 0.216 | Nafees et al, 2017 |
| Neutropenia | 114 (26%) | 20,276 | Wong et al, 2018 | 0.348 | Nafees et al, 2017 |
| Decreased platelet count | 53 (12%) | 7,789 | Konidaris et al, 2020 | 0.108 | Konidaris et al, 2021 |
| Weighted average^d^ |  | 9,692 |  | 0.152 |  |

^a^Our analysis only included and evaluated grade ≥ 3 treatment-related adverse events.

^b^Number within treatment arm: nivolumab plus ipilimumab (n = 468), and EXTREME (n = 441).

^c^Calculated as an average cost of toxicity using the weighted frequency of occurrence. This value was used in the base-case model.

**References**

Freeman K, Connock M, Cummins E, Gurung T, Taylor-Phillips S, Court R, et al. Fluorouracil Plasma Monitoring: Systematic Review and Economic Evaluation of the My5-Fu Assay for Guiding Dose Adjustment in Patients Receiving Fluorouracil Chemotherapy by Continuous Infusion. *Health Technol Assess* (2015) 19(91):1-321, v-vi. doi: 10.3310/hta19910

Konidaris G, Paul E, Kuznik A, Keeping S, Chen CI, Sasane M, et al. Assessing the Value of Cemiplimab for Adults with Advanced Cutaneous Squamous Cell Carcinoma: A Cost-Effectiveness Analysis. *Value Health* (2021) 24(3):377-87. doi: 10.1016/j.jval.2020.09.014

Nafees B, Lloyd AJ, Dewilde S, Rajan N, Lorenzo M. Health State Utilities in Non-Small Cell Lung Cancer: An International Study. *Asia Pac J Clin Oncol* (2017) 13(5):e195-e203. doi: 10.1111/ajco.12477

Wong W, Yim YM, Kim A, Cloutier M, Gauthier-Loiselle M, Gagnon-Sanschagrin P, et al. Assessment of Costs Associated with Adverse Events in Patients with Cancer. *PLoS One* (2018) 13(4):e0196007. doi: 10.1371/journal.pone.0196007
